# The Role of Admission Glucose and Inflammatory Markers in Histopathological Features of Atherosclerotic Plaques in Carotid and Femoro-Popliteal Arteries

**DOI:** 10.3390/medicina61050879

**Published:** 2025-05-12

**Authors:** Mircea Cătălin Coșarcă, Suzana Vasilica Șincaru, Emőke Horváth, Daniela Tatiana Sala, Nicolae Alexandru Lazăr, Ludovic Alexandru Szanto, Marius Mihai Harpa, Cosmin Carașcă, Gergő Ráduly, Paula Bândea, Vasile Adrian Mureșan

**Affiliations:** 1 Department of Anatomy, ‘George Emil Palade’ University of Medicine, Pharmacy, Science and Technology of Targu Mures, 540139 Targu Mures, Romania; gergo.raduly@umfst.ro; 2 Clinic of Vascular Surgery, Mures County Emergency Hospital (SCJU Târgu-Mureș), 540136 Targu Mures, Romania; 3 Department of Morphopatology, ‘George Emil Palade’ University of Medicine, Pharmacy, Science and Technology of Targu Mures, 540139 Targu Mures, Romania; 4 Department of General Surgery, ‘George Emil Palade’ University of Medicine, Pharmacy, Science and Technology of Targu Mures, 540139 Targu Mures, Romania; 5 Department of Surgery Number IV, ‘George Emil Palade’ University of Medicine, Pharmacy, Science and Technology of Targu Mures, 540139 Targu Mures, Romania; 6 Department of Forensic Medicine, ‘George Emil Palade’ University of Medicine, Pharmacy, Science and Technology of Targu Mures, 540139 Targu Mures, Romania; cosmin.carasca@umfst.ro; 7 Department of Vascular Surgery, ‘George Emil Palade’ University of Medicine, Pharmacy, Science and Technology of Targu Mures, 540139 Targu Mures, Romania

**Keywords:** atherosclerosis, glucose levels, inflammatory markers, neutrophil-to-lymphocyte ratio, platelet-to-lymphocyte ratio, lymphocyte-to-monocyte ratio, cardiovascular disease

## Abstract

*Background and Objectives*: Atherosclerosis is a chronic inflammatory disease significantly contributing to cardiovascular morbidity and mortality. This study primarily aims to evaluate the role of baseline blood glucose levels and inflammatory markers in the histopathological features of atherosclerotic plaques in the carotid and femoro-popliteal arteries. *Materials and Methods*: In this retrospective, observational, and monocentric study, 165 patients diagnosed with infrainguinal peripheral arterial disease or carotid artery disease hospitalized in the Vascular Surgery Clinic, between January 2019 and December 2023, were included. From the electronic database of the hospital, we documented demographic data, cardiovascular comorbidities, including hypertension, atrial fibrillation, ischemic heart disease, and chronic heart failure, as well as chronic kidney disease, diabetes, and prevalent risk factors such as active smoking, dyslipidemia, and obesity. Additionally, we recorded the arterial site from which the atherosclerotic plaque was obtained, along with laboratory data obtained at the time of admission prior to the surgery. The patients were divided into “Carotid Artery” and “Femoro-Popliteal Axis” based on anatomical location. *Results*: A greater prevalence of male patients (*p* = 0.008) and dyslipidemia (*p* = 0.002) was found in the group with atherosclerotic plaques from the femoro-popliteal axis. Laboratory data also showed increased lymphocyte (*p* = 0.020) and PLT (*p* = 0.028) levels in this group. There was no significant difference in the types of atherosclerotic plaques between the two patient groups. However, those in the Carotid Artery group showed a higher rate of antiaggregant treatment and a reduced incidence of dual therapy (*p* < 0.001). The Spearman correlation analysis revealed a positive correlation between baseline glucose levels and NLR (r = 0.402, *p* < 0.001), MLR (r = 0.217, *p* = 0.005), PLR (r = 0.306, *p* < 0.001), and LGI (r = 0.693, *p* < 0.001). Furthermore, the predictive roles of glucose, NLR, MLR, and LGI were assessed through multivariate analysis. Consequently, elevated baseline values of the parameters above were associated with unstable atherosclerotic plaques, independent of demo-graphic data, standard cardiovascular risk factors, site of artery harvest, and chronic vascular treatments at the time of admission (for all *p* < 0.05). *Conclusions*: This study highlights the significant relationships between glucose levels and various inflammatory markers in patients with different histopathological diagnoses of atherosclerotic plaques. Additionally, elevated glycemic and systemic inflammation biomarkers were associated with unstable atherosclerotic plaque, independent of demographic data, comorbidities, cardiovascular risk factors, anatomical artery harvest, and vascular chronic medication at the time of admission.

## 1. Introduction

Atherosclerosis, a chronic inflammatory disease of the arteries, remains a leading cause of cardiovascular morbidity and mortality worldwide [1]. Despite advances in medical research and treatment strategies, understanding the underlying mechanisms of atherosclerotic plaque formation and progression is crucial for improving patient outcomes [2]. It is well established that inflammation plays a significant role throughout the life cycle of atherosclerotic plaques [3,4,5,6,7]. Recently, various prognostic tools linked to the progression of plaques and adverse outcomes in patients with carotid atherosclerosis or peripheral artery disease (PAD) have been proposed and analyzed [8,9,10,11,12]. To date, there has been significant interest in analyzing inflammatory cytokines like interleukin-1 (IL-1), interleukin-6 (IL-6), tumor necrosis factor-α (TNF-α), and C-reactive protein (CRP). Their predictive role in atherosclerotic plaque progression has been established [3,4,9,11]. Nevertheless, the aforementioned biomarkers are expensive or unavailable in many medical centers and are not routinely used in current medical practice. As a result, inflammatory biomarkers obtained from the total counts of neutrophils, monocytes, lymphocytes, and platelets have recently been proposed due to their accessibility and affordability.

Thus, neutrophil-to-lymphocyte ratio (NLR), monocyte-to-lymphocyte ratio (MLR), and the platelet-to-lymphocyte ratio (PLR) have received considerable interest as potential indications of systemic inflammation and immune response in atherosclerosis [13,14]. Higher values are reflective of the body’s inflammatory state and have been associated with various cardiovascular events, including myocardial infarction, stroke, and peripheral artery disease [14,15,16,17,18,19,20,21,22].

Additionally, a significant challenge for specialists is managing diabetic patients with atherosclerotic disease due to the distinct microstructural changes in atherosclerotic plaques seen in these individuals [23,24,25,26]. At the iliofemoral site, diabetic patients tend to present calcified plaques and experience poorer outcomes after lower-limb surgical re-vascularization [23]. Conversely, a study by Scholtes et al. [27], which histologically examined 1455 carotid plaques, showed no significant difference in inflammatory chemokines, cytokines, or plaque characteristics between diabetic and non-diabetic patients. Therefore, the conflicting findings about the impact of diabetes and, by extension, blood glucose levels on the type and progression of atherosclerotic plaques and systemic inflammation highlight the need for further research for improved understanding.

This study primarily aims to evaluate the role of baseline blood glucose levels and inflammatory markers in the histopathological features of atherosclerotic plaques in the carotid and femoro-popliteal arteries. Furthermore, this study will investigate the relationship between glucose levels and biomarkers, as well as analyze the risk factors associated with the histopathological characteristics of atherosclerotic plaques.

## 2. Materials and Methods

### 2.1. Study Design

The current study is a retrospective, observational, monocentric study in which all patients diagnosed with infrainguinal peripheral arterial disease or carotid artery disease hospitalized in the Vascular Surgery Clinic, Emergency County Hospital Târgu Mureș, between January 2019 and December 2023, are included. Patients receiving medication therapy or endovascular treatment, as well as those diagnosed with PAD from whom no atherosclerotic plaque was obtained during surgery, were excluded. Additionally, we excluded patients with septic conditions, high inflammatory status, and those with COVID-19 due to their adverse effect on systemic inflammation and, consequently, on the inflammatory biomarkers examined in this study [28,29,30]. Additionally, patients for whom the atherosclerotic plaque samples could not be analyzed histologically, as well as those in whom we could not identify all relevant information tracked in the hospital’s electronic database, were excluded from the analysis. Finally, a total of 165 patients were enrolled in the current study, with a mean age of 65.68 ± 8.58 years; among these, 119 patients (72.12%) were male and 46 patients (27.88%) were female, as detailed in Table 1. Information regarding comorbidities, risk factors, the site of arterial harvest, the type of atherosclerotic plaque, and chronic medications at the time of admission is presented in Table 1. The patients were divided into “Carotid Artery” and “Femoro-Popliteal Axis” based on anatomical location.

### 2.2. Data Collection

From the electronic database of the hospital, we documented demographic data, cardiovascular comorbidities, including hypertension, atrial fibrillation, ischemic heart disease, and chronic heart failure, as well as chronic kidney disease, diabetes, and prevalent risk factors such as active smoking, dyslipidemia, and obesity. Additionally, we recorded the arterial site from which the atherosclerotic plaque was obtained, along with laboratory data obtained at the time of admission prior to the surgery. Concerning chronic medication, we quantified monotherapy with the antiaggregant or anticoagulant, respectively, and dual therapy that patients were undergoing at the time of admission. Based on the number of neutrophils, lymphocytes, monocytes, and platelets, titrated pre-operatively, we calculated the following inflammatory biomarkers:

- NLR = neutrophils/lymphocytes
- MLR = monocytes/lymphocytes
- PLR = platelets/lymphocytes

Additionally, we calculated the leukocyte glucose index (LGI) using the previously published formula from the total leukocyte count and glucose values [31].

### 2.3. Histopathological Characterization of Atherosclerotic Plaque

Atherosclerotic plaque specimens obtained during surgical procedures underwent a series of histopathological preparation steps. Initially, the tissue fragments were fixed in 4% formaldehyde for at least 24 h. Following fixation, an automated tissue processor prepared the samples in paraffin blocks. After this, thin sections were meticulously cut from these paraffin-embedded samples and stained utilizing the standard hematoxylin and eosin method. Two medical examiners independently evaluated and scored the gross pathological findings observed during autopsies. The degree of atherosclerosis was determined through histopathological examination, in accordance with the histological classification system established by the American Heart Association’s Committee on Vascular Lesions of the Council on Arteriosclerosis [32]. This grading system encompasses the following lesion types, which were noted in our study: atheroma (type IV), fibroatheroma, calcified lesion, fibrotic lesion (type V), and advanced lesion with surface defect, hemorrhage, hematoma, or thrombotic deposit (type VI).

### 2.4. Study Endpoint

The primary endpoint of the present study is unstable atherosclerotic plaque, characterized as type VI atherosclerotic plaque in accordance with the aforementioned AHA classification. We examined the role of systemic inflammation and risk factors in developing unstable atherosclerotic plaque. In a secondary analysis, we investigated the relationship between baseline glucose levels and systemic inflammatory status.

### 2.5. Statistical Analysis

The SPSS software for Mac OS version 29.0.2.0 was utilized for the statistical analysis (SPSS, Inc., Chicago, IL, USA). Laboratory data, age, and inflammatory biomarkers are presented as mean values ± standard deviations (SDs). To compare differences between dichotomous variables concerning the anatomical locations of the harvested atherosclerotic plaque, the Chi-square test was employed. Furthermore, the Mann–Whitney U test and Student’s *t*-test were applied for continuous variables. The relationship between baseline glucose levels and inflammatory biomarkers was evaluated using Spearman correlation. Additionally, to analyze differences in the values of white blood cells (WBCs), glucose, NLR, MLR, PLR, and LGI, while comparing the types of atherosclerotic plaques individually and based on the anatomical location from which they were obtained, a two-way ANOVA with Bonferroni multiple comparisons test was implemented. The appropriate cut-off values for WBCs, glucose, NLR, MLR, PLR, and LGI were established through ROC curve analysis based on the Youden index. Binary logistic regression was employed to examine the predictive factors associated with unstable atherosclerotic plaque. We expressed the odds ratio (OR) for each 1 standard deviation increase in the baseline values of the aforementioned variables. Furthermore, three distinct adjustment models were utilized to assess the associations between glucose levels, inflammatory biomarkers, and unstable atherosclerotic plaque. Specifically, Model 1 incorporates age and sex; Model 2 includes age, sex, and cardiovascular risk factors (diabetes, hypertension, ischemic heart disease, chronic kidney disease, active smoking, obesity); and Model 3 encompasses age, sex, cardiovascular risk factors, artery site of harvest, and treatment. All tests were two-tailed, and a *p*-value of less than 0.05 was deemed statistically significant.

## 3. Results

In the present study, a total of 165 atherosclerotic plaques were collected and subjected to analysis. Among these, 119 atherosclerotic plaques (72.12%) were acquired following carotid endarterectomy, while 46 atherosclerotic plaques (27.88%) were obtained from the femoro-popliteal axis. Concerning the histological classification of atherosclerotic plaques, 20 plaques (12.12%) were categorized as type IV, 93 plaques (56.36%) were categorized as type V, and 52 plaques (31.52%) were categorized as type VI (Table 1). About the cardiovascular comorbidities, hypertension was observed in 141 patients (85.45%), ischemic heart disease was noted in 96 patients (58.18%), and chronic heart failure was identified in 48 patients (29.09%). Furthermore, 54 patients (32.72%) were diagnosed with diabetes, 61 patients (36.96%) were identified as active smokers, 44 patients (26.67%) presented with dyslipidemia, and 37 patients (22.52%) were categorized as obese (Table 1).

Additionally, we noted a greater prevalence of male patients (*p* = 0.008) and dyslipidemia (*p* = 0.002) in the group with atherosclerotic plaques from the femoro-popliteal axis. Laboratory data also showed increased lymphocyte (*p* = 0.020) and PLT (*p* = 0.028) levels in this group. There was no significant difference in the types of atherosclerotic plaques between the two patient groups. However, those in the Carotid Artery group showed a higher rate of antiaggregant treatment and a reduced incidence of dual therapy (*p* < 0.001) (Table 2).

**Figure 1:**
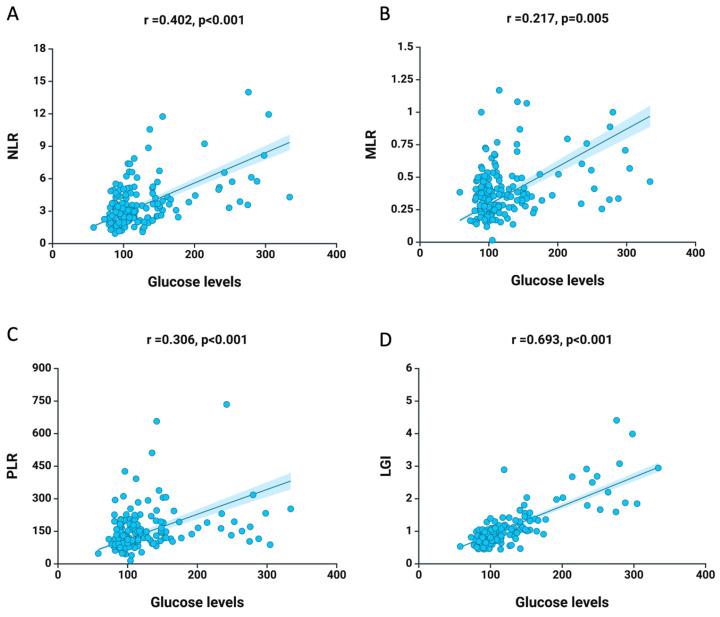
Graphical representation of the association between glucose levels at baseline and (**A**) NLR, (**B**) MLR, (**C**) PLR, and (**D**) LGI. The blue dots represent the variable values of the patients, and the blue line with the blue area represents the best-fit line and 95% confidence interval.

The Spearman correlation analysis revealed a positive correlation between glucose levels and inflammatory biomarkers. Figure 1 illustrates a strong positive correlation between baseline glucose levels and NLR (r = 0.402, *p* < 0.001), MLR (r = 0.217, *p* = 0.005), PLR (r = 0.306, *p* < 0.001), and LGI (r = 0.693, *p* < 0.001). This indicates that glycemia levels are linked to systemic inflammatory status, highlighting its significance in atherosclerotic processes.

**Figure 2:**
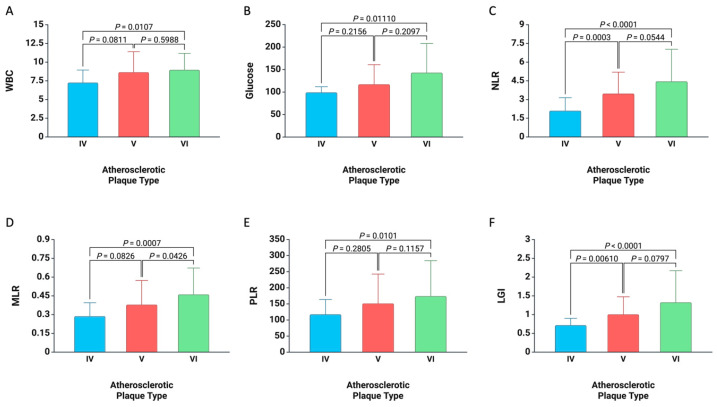
Graphical representation illustrating the differences associated with the types of atherosclerotic plaques and the baseline values for (**A**) WBCs, (**B**) Glucose, (**C**) NLR, (**D**) MLR, (**E**) PLR, and (**F**) LGI, in the entire cohort of patients.

In terms of baseline inflammatory biomarker values linked to specific types of atherosclerotic plaque, it was found that patients with type V (*p* = 0.0003) and type VI plaques (*p* < 0.0001) exhibited higher NLR values than those with type IV plaques. Similar discrepancies were noted with LGI. Additionally, for MLR, patients with type VI plaques had elevated values in comparison to those with type V (*p* = 0.0426) and type IV plaques (*p* = 0.0007). Moreover, concerning WBCs, glucose, and PLR, those with type VI plaques showed greater values compared to individuals with type IV plaques (all *p* < 0.05) (Figure 2).

**Figure 3:**
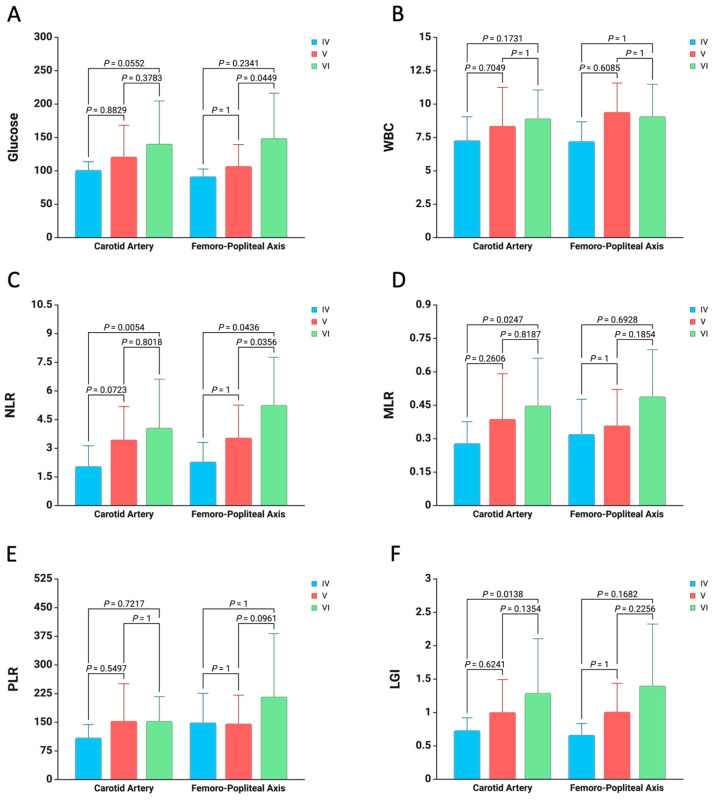
Comparison of baseline values for glucose (**A**), WBC (**B**), NLR (**C**), MLR (**D**), PLR (**E**), and LGI (**F**) in relation to atherosclerotic plaque type and artery harvest site. The columns show mean values along with standard deviations.

Furthermore, to enhance the reliability of the statistical analysis, we stratified the values of inflammatory biomarkers according to plaque type and artery harvest site in order to examine the distinctions between systemic inflammation and the remodeling of carotid or femoropopliteal atherosclerotic plaques. As illustrated in Figure 3, regarding carotid atherosclerotic plaques, elevated values of NLR (*p* = 0.0054), MLR (*p* = 0.0247), and LGI (*p* = 0.0138) are observed in patients exhibiting type VI atherosclerotic plaques when compared to those presenting with type IV atherosclerotic plaques. With respect to the femoropopliteal axis, an increased baseline value of glucose (*p* = 0.0449) and NLR (*p* = 0.0356) was noted in patients with type VI atherosclerotic plaques in comparison to their counterparts with type V atherosclerotic plaques, in addition to a higher NLR value (*p* = 0.0436) in patients with type VI relative to those with type IV atherosclerotic plaques. No significant differences were identified concerning WBC and PLR values across all types of atherosclerotic plaques (Figure 3).

At ROC Curve Analysis, a significant association was observed between baseline values for glucose (AUC: 0.614, *p* = 0.024), NLR (AUC: 0.667, *p* < 0.001), MLR (AUC: 0.651, *p* = 0.001), PLR (AUC: 0.625, *p* = 0.006), and LGI (AUC: 0.650, *p* = 0.002) and unstable atherosclerotic plaque in all patients. Consequently, an optimal cut-off value of 134.75 (42.3% Sensitivity and 82.3% Specificity) for glucose, 2.79 (80.8% Sensitivity and 49.6% Specificity) for NLR, 0.29 (82.7% Sensitivity and 43.4% Specificity) for MLR, 122.08 (71.2% Sensitivity and 55.8% Specificity) for PLR, and 0.95 (65.4% Sensitivity and 62.8% Specificity) for LGI was recorded (Table 3).

In binary regression analysis, the presence of atrial fibrillation (OR 3.71, *p* = 0.010) and elevated baseline values for glucose (OR: 1.73, *p* = 0.002), NLR (OR: 1.81, *p* = 0.002), MLR (OR: 1.89, *p* = 0.007), and LGI (OR: 1.84, *p* = 0.001) were linked to unstable atherosclerotic plaque in all patients (Table 4).

Furthermore, the predictive roles of glucose, NLR, MLR, and LGI were assessed through multivariate analysis. Consequently, elevated baseline values of the parameters above were associated with unstable atherosclerotic plaques, independent of demographic data, standard cardiovascular risk factors, site of artery harvest, and chronic vascular treatments at the time of admission (for all *p* < 0.05) (Table 5).

## 4. Discussion

The findings from this study provide a comprehensive analysis of the relationships between glucose levels and various inflammatory markers in patients with different histopathological diagnoses of atherosclerotic plaques, focusing on unstable atherosclerotic plaques. This study advances the field by reinforcing the critical role of glycemic control in modulating systemic inflammation and its implications for atherosclerotic disease progression. The strong correlations between glucose levels at baseline and NLR, MLR, PLR, and LGI highlight a direct relationship between hyperglycemia and heightened systemic inflammation. These findings align with prior studies while adding novel insights by exploring specific plaque types and their associated inflammatory profiles. Early identification of high-risk patients using these markers could guide targeted interventions, including tighter glycemic control, anti-inflammatory therapies, and lifestyle modifications.

An important discovery of this study is the positive association between glucose levels and NLR, a widely recognized indicator of systemic inflammation [33,34]. It has been linked to a negative outlook in different cardiovascular illnesses [35,36,37,38]. This discovery is consistent with prior research showing the inflammatory effects of high blood sugar levels. Thus, a study conducted by Chen et al. [39] revealed a positive association between glucose levels and increased NLR in individuals diagnosed with acute coronary syndrome. This suggests that hyperglycemia intensifies inflammatory reactions in these patients. Another study conducted by Bajari et al. [40] similarly found that an increased NLR can be used to predict negative outcomes in patients with acute myocardial infarction. Furthermore, the strong associations between glucose levels and PLR emphasize the function of hyperglycemia in stimulating inflammation and platelet activation [39]. PLR, similar to NLR, serves as a marker for widespread inflammation and has been associated with worse outcomes in cardiovascular conditions [40]. The current study observed a positive correlation between the baseline glucose levels and PLR value (r = 0.306, *p* < 0.001). This association is consistent with the findings of a study by Li et al. [41], which demonstrated that elevated PLR is an independent predictor of major adverse cardiovascular events in patients with coronary artery disease. The study also emphasized the importance of PLR as a marker of inflammation and its potential utility in risk stratification [41]. The relatively weak parallels between glucose levels and plaque location suggest that hyperglycemia is important in modulating inflammatory responses; their impact may not be strongly influenced by the anatomical location of the atherosclerotic plaques [13,42].

Concerning the distinction between carotid and femoral atherosclerotic plaques, Herisson et al. [43] noted a lower prevalence of types IV and V in the femoral artery compared to the carotid artery (7% versus 75%, *p* < 0.001), along with a higher prevalence of types VII and VIII (according to the AHA modified classification) in contrast to carotid plaques (93% versus 25%, *p* < 0.001). Furthermore, Cunnane et al. [44] conducted an analysis of the mechanical properties and composition of 24 carotid and 16 femoral atherosclerotic plaques. The authors noted a significantly higher stretch at failure (*p* = 0.012), greater strength (*p* < 0.001), and increased stiffness (*p* = 0.002) of carotid plaques in comparison to femoral plaques. Contrary to findings reported in the literature, this study found no differences in atherosclerotic plaque types between the carotid and femoral arteries. This discrepancy may be attributed to the limited cohort size analyzed in previous studies, which did not account for the influence of confounding factors on the type of atherosclerotic plaque.

Numerous recently published articles have demonstrated that inflammatory biomarkers derived from complete blood counts are associated with carotid atherosclerosis [45,46,47]. Consistent with our study’s findings, Liao et al. [45] noted through logistic regression analysis that the PLR and NLR were correlated with carotid atherosclerosis. Furthermore, Corriere et al. [46] identified that an NLR value exceeding 2.4 was associated with carotid plaques (*p* < 0.001). In the present study, an elevated baseline value of NLR (OR: 1.81, *p* = 0.002), but not PLR (OR: 1.34, *p* = 0.083), was associated with unstable atherosclerotic plaques in all patients.

Overall, our findings highlight the critical importance of glycemic control in managing systemic inflammation and improving clinical outcomes in patients with atherosclerotic disease. Monitoring and controlling blood glucose levels can help reduce inflammation and potentially prevent adverse cardiovascular events. Future research should focus on elucidating the underlying mechanisms of these associations and evaluating the efficacy of targeted interventions in reducing inflammation and improving prognosis in patients with hyperglycemia and atherosclerosis.

While our analysis provides valuable insights, several limitations inherent in the current study must be addressed. Primarily, the current study is retrospective and monocentric, having observed patients throughout their hospitalization. Future research endeavors should incorporate multicenter, prospective studies to evaluate and corroborate our findings, allowing for generalization to the broader population. A notable limitation of this study is the lack of data concerning statins and antidiabetic medications, which can significantly affect atherosclerotic plaque remodeling and progression. Additionally, not all patients had available CRP values in the hospital’s electronic database, which hindered their inclusion in this research. Future investigations should strive to collect CRP values at the time of admission and during subsequent follow-ups to assess the correlation between CRP levels and the risk of restenosis following surgical revascularization. Moreover, while our study reported the types of atherosclerotic plaques according to the AHA classification, we could not obtain complete information regarding the level of inflammation at the plaque site, neovascularization, and microstructural characteristics of the plaques for all patients. Prospective studies that utilize standardized data collection methods and control for confounding variables would yield a more comprehensive examination of these issues.

## 5. Conclusions

This study highlights the significant relationships between glucose levels and various inflammatory markers in patients with different histopathological diagnoses of atherosclerotic plaques. Elevated glucose levels at admission were strongly related to increased NLR, MLR, PLR, and LGI, emphasizing the link between hyperglycemia and systemic inflammation. Additionally, elevated glycemic and systemic inflammation biomarkers were associated with unstable atherosclerotic plaque, independent of demographic data, comorbidities, cardiovascular risk factors, anatomical artery harvest, and vascular chronic medication at the time of admission.

## Figures and Tables

**Table 1:** Characterization of participants enrolled in the current study.

Variables		All Patients *n* = 165
Age, mean ± SD		65.68 ± 8.58
Male, no. (%)		119 (72.12%)
Female, no. (%)		46 (27.88%)
Comorbidities and Risk factors, no. (%)		
Hypertension		141 (85.45%)
Atrial Fibrillation		18 (10.9%)
Diabetes		54 (32.72%)
Ischemic Heart Disease		96 (58.18%)
Chronic Heart Failure		48 (29.09%)
Chronic Kidney Disease		11 (6.67%)
Active Smoking		61 (36.96%)
Dyslipidemia		44 (26.67%)
Obesity		37 (22.42%)
Artery Site Harvest and Atherosclerotic Type, no. (%)		
Carotid Artery		119 (72.12%)
Femoro-Popliteal Axis	Common Femoral Artery	19 (11.51%)
	Superficial Femoral Artery	22 (13.33%)
	Popliteal Artery	5 (3.03%)
Atherosclerotic Plaque	Type IV	20 (12.12%)
	Type V	93 (56.36%)
	Type VI	52 (31.52%)
Chronic Medication, No. (%)		
No treatment, no. (%)		9 (5.45%)
Only Anticoagulant, no. (%)		7 (4.24%)
Only Antiaggregant, no. (%)		68 (41.21%)
Double Therapy, no. (%)		81 (49.1%)

**Table 2:** Demographic data, comorbidities, risk factors, atherosclerotic plaque characteristics, laboratory data, and chronic medication based on the artery site harvesting.

Variables		Carotid Artery *n* = 119	Femoro-Popliteal Axis *n* = 46	*p* Value
Age, mean ± SD		66.33 ± 7.96	64.02 ± 9.91	0.117
Male, no. (%)		79 (66.38%)	40 (86.95%)	0.008
Female, no. (%)		40 (33.62%)	6 (13.05%)	
Comorbidities and Risk factors, no. (%)				
Hypertension		104 (87.39%)	46 (100%)	0.255
Atrial Fibrillation		11 (9.24%)	7 (15.22%)	0.270
Diabetes		40 (33.61%)	14 (30.43%)	0.696
Ischemic Heart Disease		66 (55.46%)	30 (65.22%)	0.255
Chronic Heart Failure		30 (25.21%)	18 (39.13%)	0.078
Chronic Kidney Disease		10 (8.40%)	1 (2.17%)	0.150
Active Smoking		47 (39.49%)	14 (30.43%)	0.322
Dyslipidemia		24 (20.17%)	20 (43.48%)	0.002
Obesity		28 (23.53%)	9 (19.56%)	0.629
Atherosclerotic Plaque	Type IV	16 (13.44%)	4 (8.69%)	0.659
	Type V	67 (56.30%)	26 (56.52%)	
	Type VI	36 (30.25%)	16 (34.78%)	
Laboratory Data, mean ± SD				
Hemoglobin g/dL		13.53 ± 1.71	12.91 ± 2.46	0.094
Hematocrit %		40.39 ± 4.92	39.09 ± 7.11	0.273
WBC		8.41 ± 2.61	9.03 ± 2.21	0.075
Creatinine (mg/dL)		0.98 ± 0.33	0.89 ± 0.19	0.137
BUN (mg/dL)		41.29 ± 20.38	34.74 ± 10.48	0.159
Glucose (mg/dL)		125.18 ± 51.92	118.44 ± 48.94	0.114
Neutrophils × 10^3^/uL		5.63 ± 2.35	6.25 ± 1.97	0.423
Lymphocytes × 10^3^/uL		1.85 ± 0.71	1.90 ± 0.65	0.020
Monocyte × 10^3^/uL		0.65 ± 0.28	0.69 ± 0.24	0.450
PLT × 10^3^/uL		240.72 ± 82.86	284.61 ± 114.83	0.028
NLR		3.45 ± 2.02	4.04 ± 2.21	0.121
MLR		0.39 ± 0.21	0.40 ± 0.19	0.518
PLR		147.66 ± 82.72	172.47 ± 119.63	0.246
LGI		1.07 ± 0.62	1.08 ± 0.62	0.685
Chronic Medication, No. (%)				
No treatment, no. (%)		4 (3.36%)	5 (10.87%)	<0.001
Only Anticoagulant, no. (%)		2 (1.68%)	5 (10.87%)	
Only Antiaggregant, no. (%)		65 (54.62%)	3 (6.52%)	
Double Therapy, no. (%)		48 (40.33%)	33 (71.74%)	

**Table 3:** Characteristic ROC Curve Analysis regarding the WBC, glucose, NLR, MLR, PLR, and LGI levels at baseline and unstable atherosclerotic plaque in all patients.

Variables	Cut-Off	AUC	Std. Error	95% CI	Sensitivity	Specificity	*p* Value
Unstable Atherosclerotic Plaque							
WBC	9.03	0.592	0.048	0.499–0.685	53.8%	68.1%	0.053
Glucose	134.75	0.614	0.050	0.515–0.712	42.3%	82.3%	0.024
NLR	2.79	0.667	0.043	0.582–0.752	80.8%	49.6%	<0.001
MLR	0.29	0.651	0.045	0.562–0.740	82.7%	43.4%	0.001
PLR	122.08	0.625	0.046	0.535–0.715	71.2%	55.8%	0.006
LGI	0.95	0.650	0.048	0.556–0.743	65.4%	62.8%	0.002

**Table 4:** Binary regression analysis of the comorbidities, risk factors, and baseline value of laboratory data and unstable atherosclerotic plaque.

Variables	Unstable Atherosclerotic Plaque		
	OR	95% CI	*p* Value
Female	0.98	0.46–2.09	0.968
Atrial Fibrillation	3.71	1.36–10.17	0.010
Ischemic Heart Disease	1.80	0.88–3.67	0.106
Diabetes	1.61	0.79–3.25	0.185
Active Smoking	1.45	0.72–2.89	0.300
Obesity	2.11	0.97–4.56	0.057
WBC	1.25 *	0.91–1.73	0.184
Glucose	1.73 *	1.23–2.43	0.002
NLR	1.81 *	1.25–2.61	0.002
MLR	1.89 *	1.18–3.02	0.007
PLR	1.34 *	0.96–1.86	0.083
LGI	1.84 *	1.26–2.68	0.001

* OR expressed per 1 SD increase in baseline artery and vein diameter.

**Table 5:** Multivariate analysis of the baseline value of glucose, NLR, MLR, and LGI, and unstable atherosclerotic plaque.

Variables		Unstable Atherosclerotic Plaque		
		OR *	95% CI	*p* Value
Glucose	Model 1	1.74	1.22–2.48	0.002
	Model 2	1.75	1.21–2.52	0.003
	Model 3	1.58	1.06–2.35	0.023
NLR	Model 1	1.81	1.25–2.61	0.002
	Model 2	1.85	1.26–2.72	0.002
	Model 3	1.78	1.19–2.66	0.005
MLR	Model 1	1.88	1.18–3.02	0.008
	Model 2	1.82	1.13–2.95	0.014
	Model 3	1.78	1.09–2.92	0.021
LGI	Model 1	1.84	1.26–2.68	0.002
	Model 2	1.88	1.27–2.78	0.002
	Model 3	1.75	1.17–2.64	0.007

* OR expressed per 1 SD increase in baseline artery and vein diameter. Model 1: age and sex. Model 2: age, sex, CV risk factors (diabetes, hypertension, ischemic heart disease, chronic kidney disease, active smoking, obesity). Model 3: age, sex, CV risk factors (diabetes, hypertension, ischemic heart disease, chronic kidney disease, active smoking, obesity), artery site harvest, and treatment.

## Data Availability

The datasets used and/or analyzed during the current study are available from the corresponding author upon reasonable request.

## Abbreviations

The following abbreviations are used in this manuscript:

NLR	neutrophil-to-lymphocyte ratio
LMR	lymphocyte-to-monocyte ratio
PLR	platelet-to-lymphocyte ratio
LGI	leukocyte glucose index
